# Wound of the Penis from the Bite of a Horse

**Published:** 1846-07

**Authors:** 


***Wound of the Penis from the Bite of a Horse.***—The man to whom this accident happened is now a patient under M. Lenoir, at the Necker hospital. Although protected by the blouse and trousers, the animal had seized the penis with such force between its teeth, as to inflict a circular wound like that which would have resulted from the use of a cutting instrument. The skin was indeed entirely detached from the root of the organ. M. Lenoir at first thought that he might heal the wound by adhesion, but gangrene took place, and the corpora cavernosa were laid bare. A gangrenous spot had also appeared in the scrotum, which had become much stretched by the violence. Extravasation of urine had been avoided by the introduction of a catheter.—*London Med. Gaz., from Gaz. des Hopitaux.*

